# Quantitative Verification of Concrete Formwork-Striking-Time Reduction by High Blaine Ordinary Portland Cement

**DOI:** 10.3390/ma16031077

**Published:** 2023-01-26

**Authors:** Jinman Kim, Sangchul Shin

**Affiliations:** 1 Department of Architectural Engineering, Kongju National University, 1223-24 Cheonan-Daero, Cheonan City 330-717, Chungcheongnam-do, Republic of Korea; 2 Environment-Friendly Concrete Research Institute, Kongju National University, 1223-24 Cheonan-Daero, Cheonan City 330-717, Chungcheongnam-do, Republic of Korea

**Keywords:** type Ⅰ high Blaine ordinary Portland cement, type Ⅰ ordinary Portland cement, type Ⅲ rapid-hardening Portland cement, formwork striking time, concrete maturity method, early-age strength

## Abstract

Type Ⅰ high Blaine ordinary Portland cement (IHB) possesses the same composition as that of type Ⅰ ordinary Portland cement; however, due to its high fineness, IHB exhibits properties that are similar to those of type Ⅲ rapid-hardening Portland cement, which can reduce the formwork striking time. However, to date, no quantitative research results regarding the construction-time-reduction effect of IHB have been reported. Therefore, this study conducted experiments to verify the formwork-striking-time reduction effect of concrete using IHB. Considering seasonal changes, the strength-development characteristics, according to the outside air temperature, were examined by modifying the curing temperature conditions (5, 10, and 20 °C). Furthermore, the achievable reduction in the concrete formwork striking time was quantitatively determined by comparing and analyzing with the linear interpolation and maturity methods for improving the accuracy of the formwork striking time. The experimental results indicated that, compared with ordinary cement, early formwork striking is possible using IHB, due to earlier strength development. Thus, IHB was confirmed to be effective for construction-time reduction through early formwork striking, and it can be used as a sufficient substitute for expensive rapid-hardening cement in sites and weather conditions where rapid hardening is required.

## 1. Introduction

The most effective method to shorten the frame construction time of concrete is to advance the formwork striking time. Reducing the formwork striking time reduces the construction cost and the quantity of formworks needed [1,2,3]. The criterion for deciding the formwork striking time is determined by the initial strength-development characteristics of the cement. The formwork can only be removed if the strength of the placed concrete is sufficient to support the self-load and the load applied during construction [4,5]. There are various factors that determine the early strength of concrete, such as the types and classes of cement and concrete; whether an admixture is used; the site temperature; and the curing condition [6].

The Korean Concrete Standard Specification KCS 14 20 12—Formwork and Shores—defines the formwork striking time as the time required for the concrete to develop vertical and horizontal strengths of 5 and 14 MPa (or 2/3 or more of the design strength), respectively [7]. Furthermore, the shore removal time is defined as the time required for the concrete to attain 100% of its design strength. Therefore, creating an environment wherein the required strength can be rapidly attained can reduce the construction time and cost by shortening the formwork-retention period. Methods for early formwork striking include high temperature, steam curing, and changing the physical properties of concrete. However, the high-temperature and steam-curing methods are often impractical and can be applied only in extremely special cases [8,9]. By contrast, modifying the physical properties of concrete by changing the material composition of cement to assist in attaining higher early strength can render early formwork removal feasible [10,11,12,13,14].

**Figure 1:**
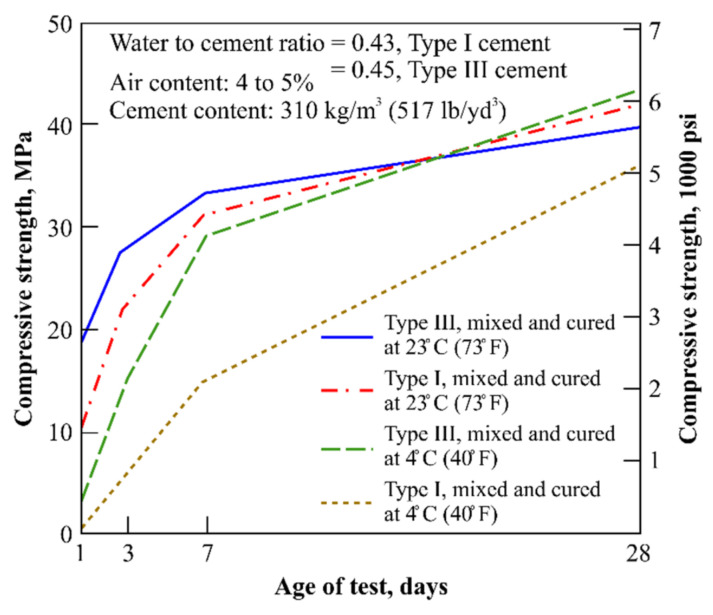
Early-age compressive strength comparison for concrete specimens fabricated with types Ⅰ and Ⅲ cement, and cured at 4 °C and 23 °C.

The compressive strength of early-age concrete is influenced by the curing temperature [15,16,17]. As shown in Figure 1, at 20 °C or higher, a compressive strength of 10 MPa is obtained after one day, whereas at approximately 4 °C, several days are required to achieve the same strength [18]. In particular, when the site temperature is below 10 °C, the strength-development rate of concrete is significantly lower than that in the standard curing environment. Thus, decreasing this difference is the main strategy for shortening the formwork striking time. As can be seen in Figure 1, the rapid hardening of the cement can advance the strength-development time. Type Ⅲ rapid-hardening Portland cement, which exhibits a high early strength and hydration heat compared to ordinary Portland cement (OPC), can be an effective solution for the delay in the formwork striking time of concrete poured at low temperatures [19,20,21,22]. However, as rapid-hardening cement is expensive and, thus, economically disadvantageous, it is rarely reflected in designs. Recently, type Ⅰ high Blaine cement (IHB), which exhibits a rapid-hardening property that is different from OPC and which is more economical than rapid-hardening cement, has been developed by a few companies in South Korea. Although IHB possesses the same composition as that of type Ⅰ OPC, its formwork striking time is reduced because it possesses a higher fineness and shows characteristics similar to those of type Ⅲ rapid-hardening Portland cement [23]. However, to date, no quantitative studies on the construction-time-reduction effect of high-fineness cement have been reported; most studies have been conducted under standard curing-temperature conditions. A quantitative approach is required, because there is no regulation for the formwork striking time of concrete structures manufactured and constructed at 10 °C or lower [24,25,26,27,28]. Therefore, this study verified the effect of using high-fineness type I cement in moderate climatic conditions of 5–20 °C.

In fact, in order to determine the formwork removal time in a concrete structure, the most reliable method is to actually take a sample from the structure and conduct a destructive test. Conducting non-destructive tests, such as ultrasound, dynamic modulus, and surface hardness verification may be the next best option [29,30,31]. However, in reality, it is difficult to conduct a non-destructive test on concrete surrounded by formwork. Therefore, in this study, a method of regression analysis of the relationship between maturity and compressive strength was adopted to determine the formwork striking time.

Based on the preceding discussion, this study aimed to experimentally verify the formwork-striking-time-reduction effect of IHB-based concrete. The control cements of this experiment were ordinary Portland cement and high-early-strength Portland cement, and the various performances of the high-fineness type I ordinary Portland cement were evaluated with control cements. The outside air temperatures were simulated in the laboratory through temperature modifications to obtain realistic strength-development characteristics according to seasonal changes, and the concrete formwork-striking-time reduction—determined by the number of days that can be achieved by applying the maturity method—was quantitatively presented. This study reports the quantitative research results regarding the construction-time-reduction effect of IHB, which is the novelty of this study.

## 2. Experimental Procedures

### 2.1. Experimental Design

Table 1 shows the experimental plan. The binders used were type Ⅰ OPC (Ⅰ), type Ⅲ rapid-hardening Portland cement (Ⅲ), and type Ⅰ high Blaine OPC (IHB), which is produced by increasing the fineness of type Ⅰ OPC. The curing temperatures were set to 5, 10, and 20 °C to create various environments corresponding to each season to which the concrete was exposed. The design strengths of the concrete specimens were 24, 27, and 30 MPa, which are frequently used in domestic construction sites.

The slump, air content, and setting time were measured in the fresh state, and the maturity was calculated by recording the temperature changes of the specimens. To identify the strength-development characteristics in the early ages after curing, the compressive strength was measured over aging periods of 0.5, 1.5, 2, and 2.5 d. In addition, compressive strength tests were conducted at 7, 14, and 28 d. The concrete was fabricated to satisfy the quality criteria of a slump of 150 ± 25 mm and an air content of 5.0 ± 1.5%. Table 2 shows the experimental mixing proportions. To examine the concrete characteristics according to the cement type, the water–cement (W/C) ratio and fine aggregate ratio were set identically for each design strength.

### 2.2. Materials

**Figure 2:**
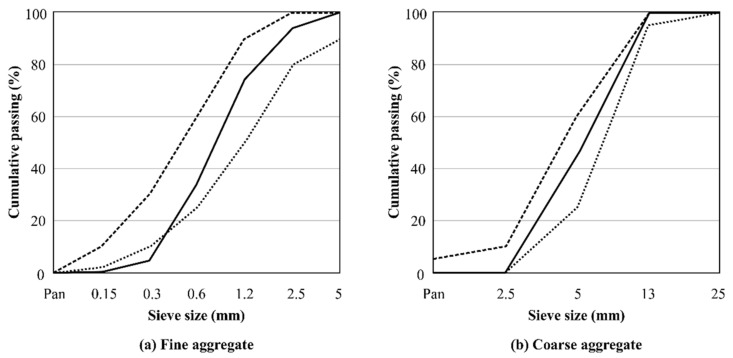
Particle-size distribution of aggregate for Korea Standard grading curve.

Table 3 shows the physical and chemical properties of the binders used in this experiment. Portland cement was obtained by adding gypsum to clinker calcined by sufficiently mixing raw materials containing lime, silica, alumina, and iron oxide as the main components in an appropriate ratio. The chemical components of ordinary Portland cement are CaO (63–67%), SiO_2_ (19–23%), Al_2_O_3_ (3–7%), Fe_2_O_3_ (1.5–4.5%), MgO (0.5–2.5%), NaO + K_2_O (0.2–1.6%), and SO_3_ (2.5–35%) [32]. As shown in Table 3, the chemical composition of IHB is similar to that of type I cement, but the difference is that the fineness of IHB is increased by more than 1000 cm^2^/g. Moreover, type III high-early-strength cement is manufactured to have more Alite(C3S) and less Belite(C2S) by increasing the amount of the raw lime material. The fineness of the type III high-early-strength cement used in this study was approximately 4800 cm^2^/g. The fineness of the IHB specimen, which was developed to increase early strength, was improved by approximately 1000 cm^2^/g more than the fineness of type Ⅰ, to approximately 4630 cm^2^/g. Aggregates of 5 mm or smaller were used for fine aggregate, and crushed aggregates of 25 mm or smaller were used for coarse aggregate. The physical properties and particle-size distribution of the aggregates are summarized in Table 4 and Figure 2. In addition, a lignin-based high-performance water-reducing agent was used for 0.5 to 0.7% of the weight of the cement, and 0.005% of air was entrained.

### 2.3. Experimental Methods

Concrete was mixed using a forced mixer (pan type: 50 l). To manufacture concrete similar to that produced by the on-site method, all aggregates and cement were poured simultaneously and dry-mixed for 1 min. Thereafter, the high-performance water-reducing agent and the air-entraining (AE) agent were mixed with the mixing water. To set the on-site pouring temperature, the temperatures of the cement, the aggregate, and the water were set to 10 °C in the 5 °C and 10 °C experiments, and to 20 °C in the 20 ℃ experiment. The specimens for measuring the compressive strength were molded with a cylinder mold of Φ100 × 200 mm, and the specimens for measuring the setting time were molded using beam molds of 100 × 100 × 400 mm. To prevent moisture evaporation, the specimens were sealed with vinyl caps and air-dried in a constant-temperature and constant-humidity chamber after pouring. All the tasks, including demolding, were performed inside the chamber to maintain the temperature.

The slump, air content, and setting time of the specimens were measured immediately after fabrication. For the measurement of setting, only the mortar was separated by wet sieving. The concrete temperature was measured every minute by installing a thermocouple at the center of each specimen, and the maturity was calculated based on these measurements. The compressive strength was measured using a universal testing machine (UTM) that could measure a load of up to 100 metric tons. The slump, air content, setting time, and compressive strength were measured according to KS F 2402 [33], KS F 2421 [34], KS F 2436 [35], and KS F 2405 [36], respectively; the Portland cement concrete slump was measured using the standard test method, content of fresh concrete was measured using the pressure method, the setting times of the concrete mixture were measured by penetration resistance, and compressive strength of concrete was measured by using the standard test method.

Regarding concrete maturity, various methods have been proposed by various researchers. Among them, the Nurse–Saul method [37] expresses the temperature history of concrete as integrated with respect to the time axis, and can be said to be the simplest maturity function. On the other hand, the method proposed by Hansen and Pedersen [38] can express the relationship between the initial strength-development rate and the curing temperature as a nonlinear relationship, but it is a relatively complex-function model. Plowman’s logarithmic function model [39] is convenient to apply in a very simple form, but has a problem of poor accuracy at low or large concrete maturity. In addition, it has been reported that the model in the form of a hyperbolic function proposed by Carino [40] has a disadvantage of low accuracy in estimating the long-term field. In this study, maturity was calculated by Equation (1) using the Nurse–Saul method suggested in ASTM C 1074 [41].

*M*(*t*) = ∑(*T_a_* − *T*_0_)∆*t*, (1)

where *M*(*t*) is the time–temperature factor at age t (degree-days or degree-hours), ∆*t* is the time interval (days or hours), *T_a_* is the average concrete temperature during the time interval (∆*t*, °C), and *T*_0_ is the datum temperature (−10 °C).

Furthermore, to investigate the time when the vertical and horizontal compressive strengths of concrete attained 5 and 14 MPa, respectively, the formwork striking time was determined using a regression analysis between the compressive strength and maturity at each age.

## 3. Results and Discussion

Table 5 shows all the experimental results, including the properties of fresh concrete, setting time, and compressive strength.

### 3.1. Properties of Fresh Concrete

The slump measurement results, according to the curing temperature and cement type, showed that every specimen satisfied the target value of 150 ± 25 mm. In general, the higher the concrete temperature and the smaller the unit quantity, the lower the consistency [17,42]; the same trend appeared in this experiment. Thus, to adjust the target slump, the amount of high-performance water-reducing agent was increased as the concrete mixing temperature and design strength increased. However, at a design strength of 30 MPa, the target slump was satisfied with some consistency, regardless of the temperature condition. The rapid-hardening cement was expected to exhibit lower workability, due to the high viscosity because of the high fineness; however, the differences in the experimental results were negligible. On the contrary, compared to type Ⅰ, IHB and type Ⅲ could achieve relatively high workability with a smaller amount of water-reducing agent.

The air content of concrete using an AE agent increases as the W/C and slump increase, and as the fineness of cement and the temperature of concrete decrease [43,44]. The air content measurement result showed that as the concrete temperature increased, the air content decreased. The air content of IHB and type Ⅲ with high fineness was lower than that of type Ⅰ. The amount of AE agent used increased slightly as the concrete temperature increased, indicating the effect of temperature. Furthermore, concrete that satisfied the target quality (5 ± 1.5%) could be fabricated in every mixture.

The temperature ranges of concrete measured immediately after mixing were 13.0–14.7 °C, 17.2–19.5 °C, and 22.7–24.5 °C at curing temperatures of 5, 10, and 20 °C, respectively. Thus, the temperature difference within each series was approximately 2 °C. Controlling the material and laboratory temperatures according to external site temperature rendered it possible for the manufactured concrete to exhibit a constant difference in pouring temperature. This temperature value can be considered as a starting temperature in the calculation of maturity.

### 3.2. Temperature History

**Figure 3:**
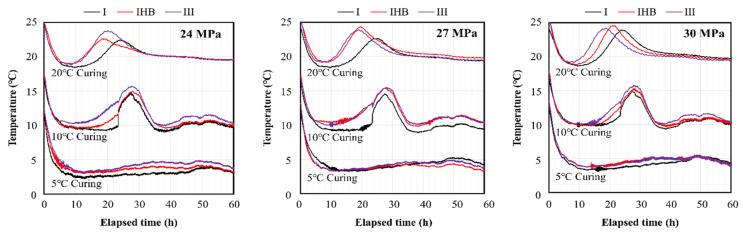
Temperature history according to curing temperature and binder type for different design strengths.

Figure 3 shows the internal temperature history of the concrete. Measurement errors occurred in some specimens. Temperature sensors embedded in concrete sometimes suffer from errors in data reading, due to the movement and bonding of materials inside the cement matrix because of the hydration reaction of cement. However, this error was not a big problem in interpreting the hydration temperature history of concrete in this study.

In the experiment with a curing temperature of 5 °C, the initial temperature of the concrete specimens was above 13 °C, which decreased continuously for approximately 10 h until the concrete specimens attained a minimum temperature of approximately 3–4 °C; thereafter, the temperature increase from the hydration reaction of cement was only approximately 2 °C, the maximum temperature was 5 °C, and the peak arrival time was 50 h. Thus, the internal temperature of concrete increased extremely slowly. Although these measurement results were obtained from small specimens, the heat of hydration of the concrete specimens was negligible, due to the effect of the ambient temperature at 5 °C. At the curing temperature of 10 °C, the temperature decreased to the chamber temperature of 10 °C and then increased slowly because of the heat of hydration, attaining a maximum temperature of 15 °C in approximately 27 h. On attaining the maximum temperature, a rapid convergence to the curing temperature of 10 °C was observed; thereafter, the temperature slightly increased and remained constant. In the experiment at 20 °C, the temperature fell below the curing temperature and then increased again. The maximum temperature was approximately 24 °C. On attaining the peak, the temperature gradually decreased and maintained the 20 °C level. Furthermore, at 20 °C, there were some variations in the maximum temperature and the maximum temperature arrival time, depending on the binder type. The heating rates and maximum temperatures of the IHB and type Ⅲ specimens were higher than those of type Ⅰ. In contrast, this difference became smaller as the curing temperature decreased. In particular, at 5 °C, the difference with respect to the cement type was marginal. Thus, the temperature history according to the binder type showed that the hydration rates of mixtures using the rapid-hardening binders IHB and type Ⅲ were faster than that of type Ⅰ specimens; however, when the curing temperature was low, the difference was insignificant.

These results confirmed that the internal temperature of the specimen drops to the ambient air temperature or below because of the effect of ambient air, despite the initial pouring temperature of concrete being higher than the curing temperature. Furthermore, as the temperature was lowered, the temperature rise was minimal, because the hydration heat of cement was offset by the ambient air temperature.

### 3.3. Setting Time

**Figure 4:**
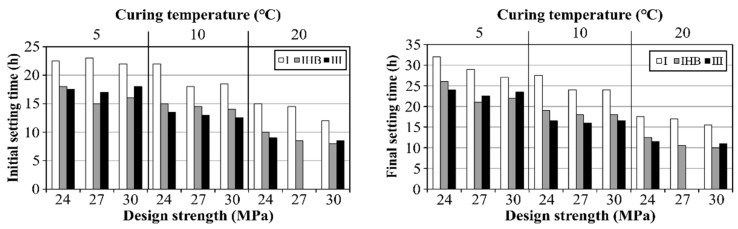
Initial and final setting times according to binder type and curing temperature.

Figure 4 shows the initial and final setting times determined from the penetration resistance value of concrete according to the cement type and design strength at each curing temperature. The initial measurement setting times of the type I specimens were 22–23 h, 18–22 h, and 12–15 h at 5, 10, and 20 °C, respectively. Furthermore, the final setting times of the same specimens were 27–32 h, 24.0–27.5 h, and 15.5–17.5 h at 5, 10, and 20 °C, respectively. To analyze the overall setting characteristics of the concrete using type Ⅰ, specimens with different design strengths were subjected to the same curing environment. The setting time decreased with an increase in design strength because the amount of reacting cement increased. Even with concrete of the same mixture, the higher the curing temperature, the faster the setting time, because the hydration reaction was promoted. The difference in curing temperature exerted a greater effect on the setting time than the design strength, and this tendency was the same with IHB and type Ⅲ specimens.

When the specimens using IHB and type Ⅲ with rapid-hardening performance were compared with the specimen using type Ⅰ under the same conditions, setting appeared to be promoted in both the initial and final times, despite differences in the temperature. Moreover, the setting times of the concrete specimens using IHB and type Ⅲ showed a decreasing trend at all curing-temperature conditions. This implies that the concrete setting time decreases with increasing design strength and curing temperature. However, the setting could be accelerated if a rapid-hardening binder was additionally used. In this experiment, as the curing temperature was high, the setting-time-reduction effect from the use of a rapid-hardening binder was greater. This suggests that the potential for the rapid response of the rapid-hardening binder can increase with an increasing ambient temperature.

**Figure 5:**
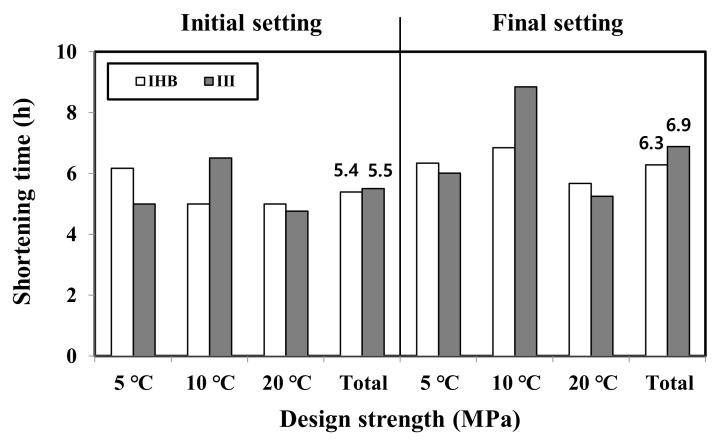
Shortening time of the IHB and type Ⅲ binders when compared with the initial and final setting times of the type Ⅰ binder.

**Figure 6:**
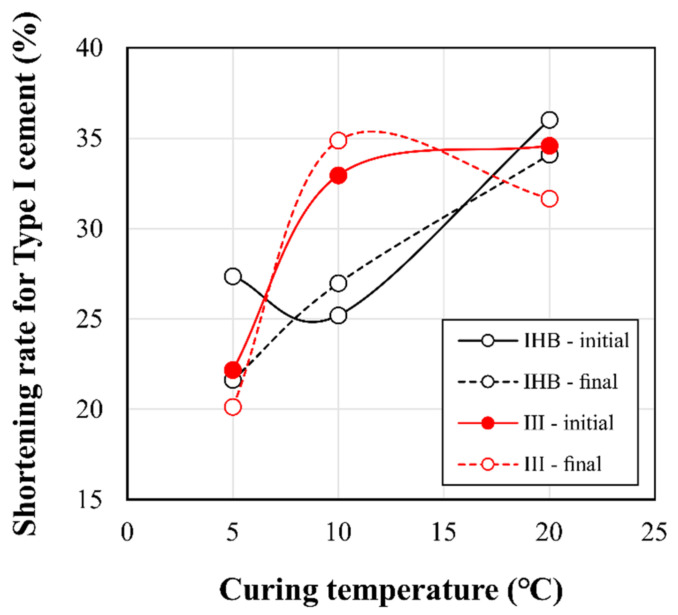
Shortening rate of the IHB and type Ⅲ binders when compared with the initial and final setting times of the type Ⅰ binder.

To examine the overall trend of the setting-time-reduction effect, the average values of the initial and final setting-time reduction using the IHB and type Ⅲ binders were as shown in Figure 5. The initial setting time was reduced by 5.4 and 5.5 h for the IHB and type Ⅲ specimens, respectively; the final setting time was reduced by 6.3 and 6.9 h for the IHB and type Ⅲ specimens, respectively. Overall, the difference in the setting time by binder type was not significant. When comparing the average values, the setting time reduction effects of the IHB and type Ⅲ binders were similar. However, at 10 °C, the setting time of the type Ⅲ specimen was faster than that of the IHB specimen, as can be observed in Figure 6. Figure 6 shows the setting-time-reduction effects of the IHB and type Ⅲ specimens in percentages, compared with that of the concrete using type Ⅰ. The setting-time-reduction rate of IHB decreased as the curing temperature decreased. By contrast, the setting time of the type Ⅲ specimen at 10 °C was higher than that of the IHB specimen, which was similar to the reduction rate at 20 °C. Therefore, it can be concluded that the lower the curing temperature, the smaller the setting-time-reduction rate of the rapid-hardening binder. Furthermore, the setting-time-reduction effects of the IHB and type Ⅲ binders exhibited slight differences with respect to the curing temperature.

These results indicate that the IHB-based concrete may exert a smaller setting-time-reduction effect than type Ⅲ in some temperature sections; however, the difference is not large. Thus, the early-strength-development property (the setting-time-reduction effect) of IHB was close to that of the type Ⅲ specimen.

### 3.4. Compressive Strength

**Figure 7:**
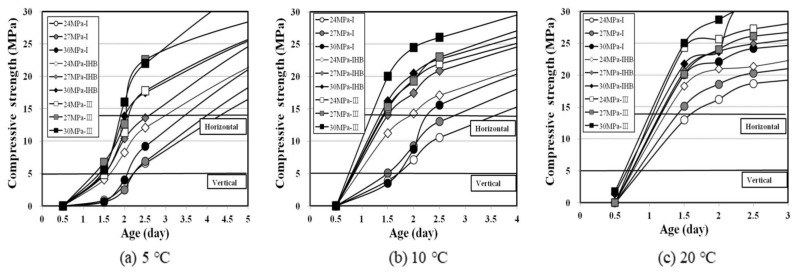
Properties of early-strength development according to binder type and curing temperature.

Figure 7 shows the early-compressive-strength expression characteristics according to the binder type, design strength, and curing temperature. The horizontal lines in Figure 7 were drawn through linear interpolation to indicate the attainment of compressive-strength values of 5 and 14 MPa, which are the criteria for vertical and horizontal formwork striking times, respectively. The comprehensive review of the strength-development characteristics during the early age showed significantly different patterns with respect to the curing temperature, which can considerably change the formwork striking time.

In the experiment at a curing temperature of 5 ℃, the type Ⅰ specimen showed a low strength of 1 MPa or lower at 1.5 d, whereas specimen types IHB and type Ⅲ showed a comprehensive strength of approximately 5 MPa, exhibiting faster hydration reaction than the type Ⅰ specimen, even at lower temperature conditions. Therefore, it was considered that the early-strength-development effect from the rapid-hardening binder and, in particular, the reduction effect of the vertical formwork striking time, could be expected. Furthermore, compared to the type Ⅲ specimen, the IHB specimen showed a larger compressive strength over the aging period of 2.5 d, and as the design strength increased, the compressive strength at the same age tended to increase slightly. Meanwhile, the higher the age, the greater the strength difference between specimens. Thus, the range of the horizontal formwork striking time became wider than that of the vertical formwork striking time.

At a curing temperature of 10 °C, the compressive strength of the type Ⅰ binder at the age of 1.5 d was close to 5 MPa. However, the compressive strength of all the specimens using rapid-hardening binders, except the 24 MPa-IHB specimen, exceeded 14 MPa, showing strength-development characteristics faster than those at the 5 °C condition. It was presumed that at 5 °C, the strength developed rapidly after the age of 1.5 d, whereas at 10 °C, the strength developed actively after 0.5 d. The vertical formwork striking of all the concrete using IHB and type Ⅲ cement was speculated to be possible within 1 d after pouring, and the horizontal formwork striking would require approximately 1.5 d; the 30 MPa-type Ⅲ-based concrete was confirmed to possess the fastest early-strength-development time.

Meanwhile, in the case of 20 °C, the compressive strength in the early age was considerably high, and most specimens showed strength values higher than 14 MPa at the age of 1.5 d, which is the criterion for horizontal formwork striking time. Particularly in the experiments at 5 °C and 10 °C, the compressive strength at the age of 0.5 d, which could not be measured, was 1.4–2.4 MPa in some specimens, confirming rapid strength-development characteristics during the early age. It is estimated that the vertical formwork of all specimens could be removed within 24 h after pouring. Moreover, because the range of the horizontal formwork striking time at 20 °C was shorter than those at other temperature conditions, the construction-time-reduction effect of the rapid-hardening binder was considered marginal.

**Figure 8:**
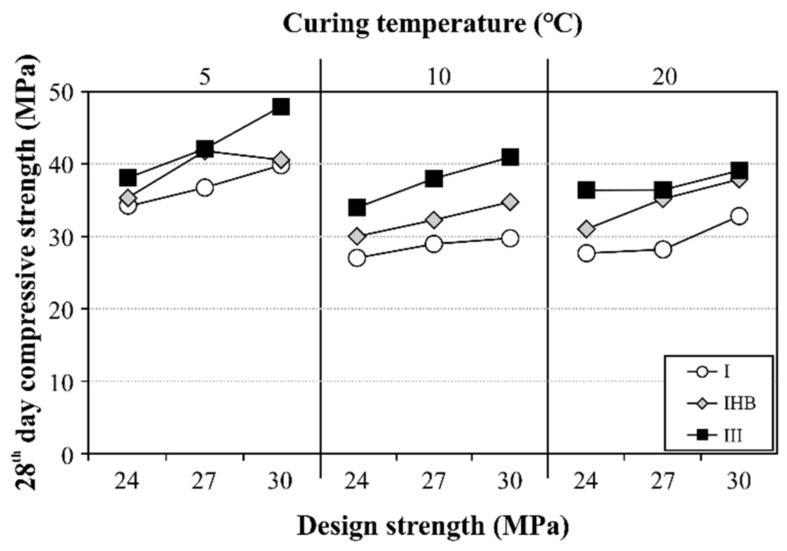
Compressive strength according to binder type and curing temperature after 28 d.

Figure 8 shows the measurement results of the compressive strength after 28 d, and the design strength was satisfied in every condition. In general, a high curing temperature is advantageous for strength in early age, because chemical reaction is promoted by hydration; however, the hydration speed decreases after approximately 7 d, which renders it disadvantageous for strength increase thereafter [45,46,47]. This characteristic was confirmed in this study. The 28 d compressive strength at 20 °C, where the strength-development rate in the early age was rapid, was similar to that of the specimen cured at 10 °C. The compressive strength at 5 °C was the highest, irrespective of the binder type. The 28 d strength values at 10 °C and 20 °C were similar; however, it can be expected that after 28 d, the compressive strength of the concrete cured at 10 °C would increase further. Moreover, the lower the curing temperature, the smaller the difference in the 28 d compressive strength between the ordinary concrete and the concrete that used the rapid-hardening binder. Meanwhile, compared to the type Ⅰ specimen, the effect of IHB on the 28 d strength was higher in all conditions, confirming the excellent performance of IHB in the mechanical properties at the age of 28 d, as well as in the reduction of the formwork striking time. However, additional review of the long-term strength and durability characteristics is necessary because the long-term strength of rapid-hardening cement becomes reversed with respect to ordinary cement.

### 3.5. Maturity

The most accurate analysis method to assess the formwork striking time is to directly examine the time when the specimen satisfies the target compressive strength by increasing the amount of specimens and setting a narrow measurement section at the early age. However, this method requires a significant amount of effort and is considered tedious. Therefore, many previous studies determined the formwork striking time using the linear interpolation method [48,49,50]. As can be observed in Figure 7, deciding the formwork striking time by linear interpolation can cause errors, based on the results of this study. At 5 °C, it was difficult to estimate the time required for the specimens that did not attain 14 MPa. At 10 °C and 20 °C, 5 MPa can be achieved between 0.5 and 1.5 d; however, the accuracy of the time prediction is limited. In addition, according to some recent studies [51,52,53], various methods to predict the compressive strength of early-age concrete have been tried, but such methods are still in the research stage. Therefore, in this study, the maturity when the target compressive strength is attained was determined by applying the maturity theory, and the formwork striking time was reviewed based on this maturity.

**Figure 9:**
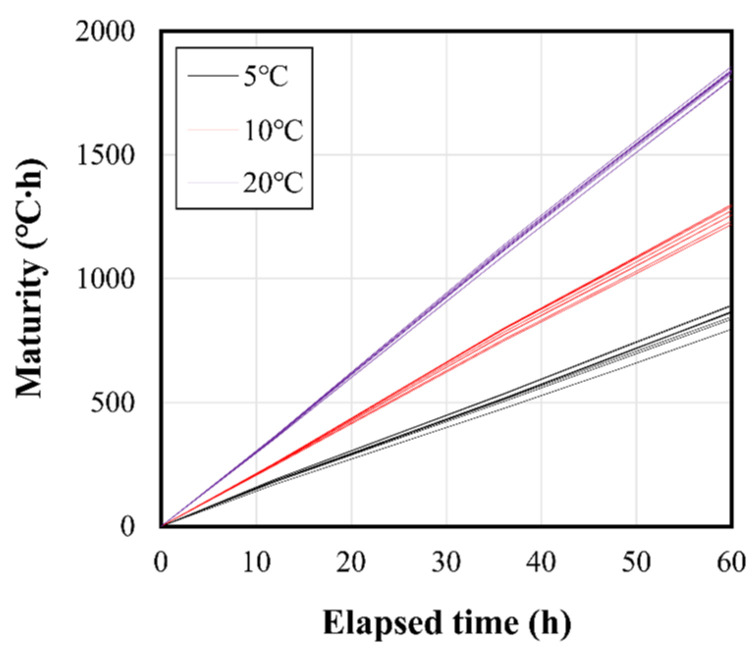
Maturity of concrete according to curing temperature.

Figure 9 shows the maturity of each specimen according to the curing temperature. As shown in Figure 3, the temperature history of the concrete specimens differs by binder type and design strength, and the maturity values were calculated in nine lines by the curing temperature series; however, the maturity lines are expressed with the same color in Figure 9, because they show similar slopes at the same temperature condition. Maturity increased faster at higher curing temperatures, and the difference in maturity increased with time. Compared to the level at 20 °C, the maturity at the same elapsed time was approximately 70% and 50% at 10 °C and 5 °C, respectively.

**Figure 10:**
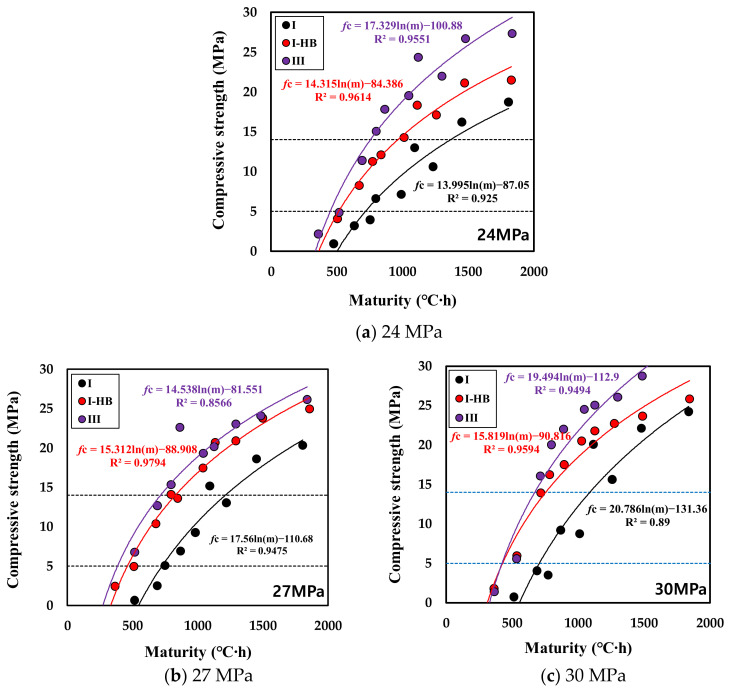
Relationship between maturity and compressive strength.

The fundamental concept of maturity is that concrete of the same mix exhibits the same strength, eve n if the curing temperature is different. Therefore, the early-strength-development characteristics can be determined by comparing the maturity according to the compressive-strength measurement of concrete and the temperature history at the strength-measurement age. In addition, from the relationship between the maturity and time, as shown in Figure 9, the elapsed time at which a certain strength level was attained can be analyzed [54,55,56]. By adopting this approach, this study represents the relationship between the maturity and the compressive strength for each design strength through logistic regression analysis, as shown in Figure 10. The maturity index shown on the X-axis of Figure 10 was calculated at strength testing times using Equation (1). In addition, the equation in the form of ⌈*f*c = a + b∙log(M)⌋ and the coefficient of determination R^2^ are shown on the graph. The specimens were analyzed for maturity and strength based on a total of 12 data of age 0.5 d, 1.5 d, 2 d, and 2.5 d at curing temperatures of 5, 10, and 20 °C. The statistical results are shown in Table 6. As a result of the regression analysis, the average difference between the linear model value and the observed value was 1.17 to 3.10. The 27 MPa-IHB test specimen showed the lowest value of 1.17, and the 30 MPa-I test specimen had the highest value of 3.10. Significance F also showed very low values (0.000345~0.00000005) in all test specimens, indicating that there was a significant correlation. From the above results, the maturity analysis based on the temperature history showed relatively good results, with a coefficient of determination of 0.90 or more under most conditions.

Upon reviewing with respect to the binder type, the type Ⅰ specimen was found to require a higher maturity to express the same level of compressive strength, whereas the type Ⅲ and IHB specimens could achieve the required strength with a lower maturity through the rapid-hardening property. This trend appeared the same in all design strength values. The maturity required to express 5 MPa, which is the criterion for vertical formwork striking, was the lowest for the type Ⅲ specimen, and that of IHB was similar to or slightly higher than that of type Ⅲ. If the type Ⅲ or IHB specimen was used instead of the type Ⅰ specimen, the maturity could be lowered by approximately 150–250 °C∙h. The difference in maturity required for horizontal formwork striking between type Ⅲ and IHB specimens increased compared to that of the vertical formwork striking time. This implies that the horizontal formwork striking time reduction effect of IHB can be lower than that of specimen type Ⅲ.

**Figure 11:**
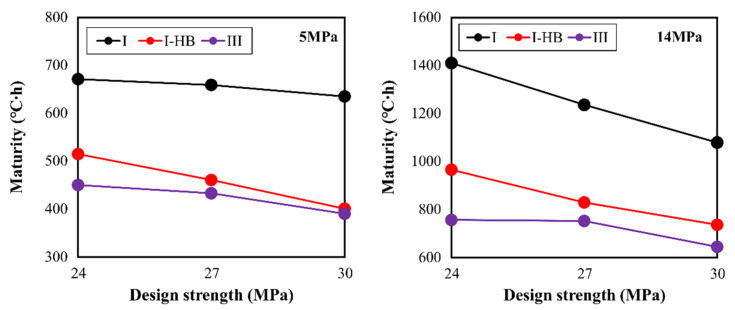
Maturity for strength development of 5 MPa and 14 MPa.

Based on these results, the maturity values required for attaining 5 and 14 MPa and the criteria for formwork removal are summarized in Figure 11. First, the maturity for expressing 5 MPa was 635–671 °C∙h for the type Ⅰ binder, 391–450 °C∙h for the type Ⅲ binder, and 401–515 °C∙h for the IHB binder. That is, compared to the specimen using type Ⅰ, those using type Ⅲ and IHB could attain 5 MPa with 65% and 70% levels of maturity, respectively. With respect to the attainment of 14 MPa, the horizontal formworks of type Ⅲ and IHB specimens could be removed at 58% and 68% of the maturity of type Ⅰ, respectively. Furthermore, the larger the design strength, the lower the maturity, because the unit binder quantity increased and the hydration reaction was promoted.

### 3.6. Determination of Demolding Time

By applying the maturity theory, the possible formwork striking time was derived in this study by substituting maturity in Figure 11 in the relation between the elapsed time and maturity in Figure 9. Table 6 shows the time required for formwork striking separately for the maturity method (M) and the generally applied linear interpolation method (L). The difference values were added to show the difference between methods M and L. The maturity method was used to obtain the elapsed time value by substituting the maturity value required to satisfy 5 and 14 MPa in each condition (Figure 11) as the y value in the linear function (y = ax + b) of the maturity elapsed time in Figure 9. The linear interpolation method was used to determine the horizontal axis values that satisfy 5 and 14 MPa in the strength-development graph in Figure 9 as the possible formwork striking time.

**Figure 12:**
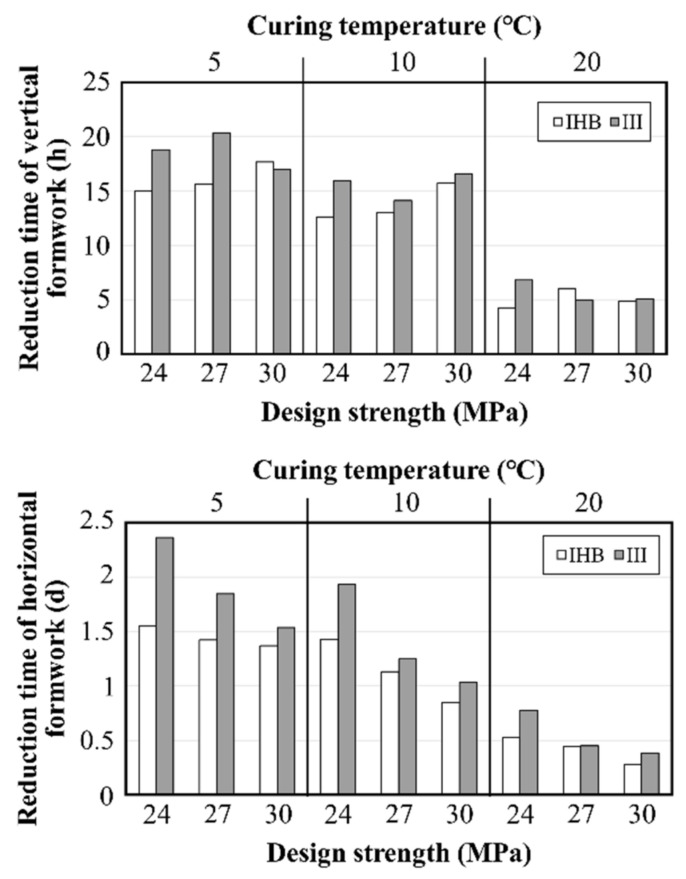
Reduction in removal time of vertical and horizontal formwork, as compared with Type I.

The analysis results showed that the difference in the formwork striking times derived by methods M and L was not large and was relatively adequate for specimens using IHB and type Ⅲ binders. Therefore, it was considered that there would be no problem in applying both the maturity method and the linear interpolation method. However, in the case of the type Ⅰ specimen, it was difficult to accurately predict the formwork striking time because the formwork striking times estimated by the maturity method at 5 °C and 10 °C were smaller than those of the linear interpolation method, and the time at 20 °C was large in some cases. Therefore, for more rational analysis, the average value of the two methods was presumed as the possible formwork striking time, and the construction-time-reduction effect from the use of a rapid-hardening binder was analyzed based on this value. Figure 12 shows the reduction in vertical and horizontal formwork striking times of concrete specimens using the IHB and type Ⅲ binders. The vertical formwork striking time of the concrete using the type Ⅰ binder was 47–52 h at 5 °C, 34–36 h at 10 °C, and 19–21 h at 20 °C. For the rapid-hardening binders IHB and type Ⅲ, the formwork striking time could be reduced by 15–20 h at 5 °C, 13–17 h at 10 °C, and 4–7 h at 20 °C. Moreover, the type Ⅲ specimen was found to exhibit a slightly higher rapid-hardening performance than IHB. In the case of the horizontal formwork, the construction-time-reduction effect of the rapid-hardening binder was verified. The lower the temperature, the larger the formwork striking-time-reduction effect. When IHB was used, the formwork striking time could be reduced by approximately 1.5 d at 5 °C and by 0.8–1.4 d at 10 °C. The reduction time of type Ⅲ was greater than that of IHB. However, at 20 °C, the reduction effect was not as large as that observed for the vertical formwork.

**Figure 13:**
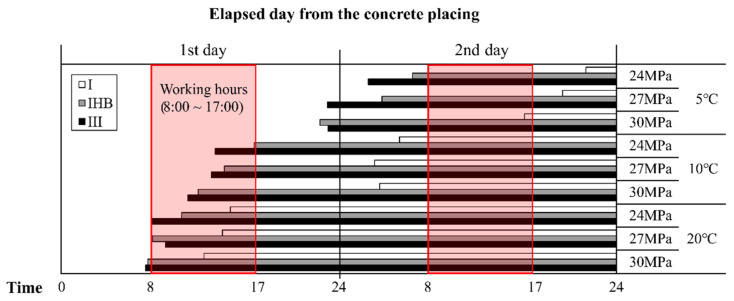
Possible time of removal of vertical form considering the on-site working time (in the case of concrete poured at 5:00 PM).

In Republic of Korea, concrete pouring work is finished by 5 PM, with the enforcement of the “Ready-mixed concrete 8–5 system,” which requires the transportation of ready-mixed concrete between 8 AM and 5 PM. Figure 13 shows the possible time of the vertical formwork striking work when the concrete-pouring finish time is assumed to be 5 PM. The section from 8 AM to 5 PM is marked by a red box as the possible work time, considering the field-work time. When type Ⅰ binder is used at a 5 °C condition, the strength for vertical formwork striking is expressed in the evening hours on the second day from the pouring day; however, when the IHB and type Ⅲ binders are used, the formwork striking can be performed from the morning of the second day. At 10 °C, the formwork striking work can be performed in the morning of the second day; however, if the rapid-hardening binder is used, the formwork striking work can be performed in the morning or afternoon of the first day. Therefore, at 5 °C and 10 °C, the vertical formwork striking-time-reduction effect of the rapid-hardening binder can be considered a one-day reduction. Meanwhile, at the 20 °C curing temperature, the formwork striking is possible on the subsequent day of pouring in all conditions. Thus, the reduction effect is not significantly different from that of lower temperature conditions. When compared with the type Ⅲ specimen, IHB possessed a smaller formwork striking-time-reduction effect. However, considering the field situation, the reduction effects of the IHB and type Ⅲ specimens on the vertical formwork striking time were considered to be similar. Consequently, the use of IHB was confirmed to be an effective method for the reduction of the construction time through early-strength development.

The comprehensive analysis of the use of IHB revealed that it enables early formwork striking, due to the faster expression of early strength compared with that of ordinary cement, and the formwork striking-time-reduction effect in low-curing-temperature conditions was particularly excellent. Furthermore, IHB is speculated to have sufficient potential as an alternative binder for rapid-hardening cement, considering that IHB possesses similar early-strength-development performance and is more economical than the rapid-hardening cement.

## 4. Conclusions

This study quantitatively verified the formwork striking-time-reduction effect of concrete using IHB and yielded the following conclusions.

1. The results from temperature history measurement revealed that the IHB and rapid-hardening cement showed rapid hydration rates and high maximum temperatures at 20 °C curing. However, this difference decreased at lower curing temperatures.
2. Compared to that of OPC, the setting time of the concrete using IHB was reduced by approximately 5.4 h and 6.3 h for the initial and final settings, respectively. Thus, the early-strength-development property of IHB was found to be close to that of the rapid-hardening cement.
3. The compressive strength at the early age was larger in the rapid-hardening cement and the mixture using IHB, and the strength difference with the OPC increased as the temperature decreased.
4. When IHB was used, the mechanical properties after 28 d were better than those of OPC. However, the long-term strength and durability characteristics should be studied further, because the long-term strength of rapid-hardening cement is generally known to be reversed with the general mix.
5. At higher curing temperatures, maturity increased more rapidly, and the maturity difference increased with time. The maturity at the same elapsed time was approximately 70% and 50% at 10 °C and 5 °C, compared to that at 20 °C.
6. The result of the regression analysis between maturity and compressive strength indicated that there is a significant correlation, with a coefficient of determination of 0.90 or more under most conditions.
7. For improved accuracy of determination of the formwork striking time, the linear interpolation method and the maturity method were compared and analyzed. The possible formwork striking time was determined using the average of these two methods.
8. Compared to ordinary cement, IHB enabled earlier formwork striking, due to earlier strength expression. In particular, below 10 °C, the construction time reduction of approximately 1 d was possible for vertical formwork and up to 2 d for horizontal formwork.

From the results of this study, IHB was confirmed to be an effective binder for construction time reduction through early formwork removal, and it can be used as a sufficient alternative to the expensive rapid-hardening cement in construction conditions that require the rapid-hardening property. However, it is considered necessary to verify the field applicability test of the mechanical properties of actually constructed concrete, because real concrete mixture can be difficult to accommodate in maturity predictions.

## Figures and Tables

**Table 1:** Experimental plan.

Factors	Levels	Test Items
Types of cement	Type Ⅰ ^(1)^, IHB ^(2)^, type Ⅲ ^(3)^	Slump Air content Setting time Temperature history and maturity Compressive strength (at 0.5, 1.5, 2, 2.5, 7, 14, and 28 d)
Curing temperature (°C)	5, 10, 20	
Design strength (MPa)	24, 27, 30	

^(1)^ type Ⅰ: type Ⅰ ordinary Portland cement, ^(2)^ IHB: type Ⅰ high Blaine cement, ^(3)^ type Ⅲ: type Ⅲ rapid-hardening Portland cement.

**Table 2:** Mixing proportions.

Design Strength (MPa)	Types of Cement	W/C (%)	S/a (%)	Unit Weight (kg/m^3^)			
				Water	Cement	Sand	Gravel
24	Type Ⅰ	48.3	47.0	160	331	837	980
	IHB	48.3	47.0	160	331	833	976
	Type Ⅲ	48.3	47.0	160	331	835	978
27	Type Ⅰ	45.5	46.5	167	367	806	963
	IHB	45.5	46.5	167	367	802	958
	Type Ⅲ	45.5	46.5	167	367	804	961
30	Type Ⅰ	42.0	46.1	165	393	791	961
	IHB	42.0	46.1	165	393	787	956
	Type Ⅲ	42.0	46.1	165	393	789	959

**Table 3:** Properties of binders.

Types	Physical Properties			Chemical Composition (%)					
	Density (g/cm^3^)	Blaine (cm^2^/g)	Soundness (mm)	CaO	SiO_2_	Al_2_O_3_	Fe_2_O_3_	MgO	Ig. loss
Type Ⅰ	3.15	3420	0.8	62.44	21.12	4.40	3.19	3.10	3.36
IHB	3.14	4630	1.0	63.28	21.40	4.36	3.16	2.77	3.20
Type Ⅲ	3.13	4790	1.0	63.09	20.99	4.50	3.29	2.77	2.23

**Table 4:** Properties of aggregate.

Types	Density (g/cm^3^)	Unit Weight (kg/L)	Absorption (%)	F. M.
Fine aggregate	2.60	1.69	0.43	2.6
Coarse aggregate	2.68	1.52	0.67	6.0

**Table 5:** Experimental results.

Curing Temp. (°C)	Design Strength (MPa)	Types of Cement	Slump (mm)	Air Content (%)	Concrete Temp. (°C)	Setting Time (h)		Compressive Strength (MPa)						
						Initial	Final	0.5 Days	1.5 Days	2 Days	2.5 Days	7 Days	14 Days	28 Days
5	24	Type Ⅰ	150	5	14.4	22.5	32	0	0.9	3.2	6.6	22	28.7	34.4
		IHB	170	5.8	14.7	18	26	0	4.1	8.2	12.1	26.6	33.6	35.3
		Type Ⅲ	150	5.8	14.5	17.5	24	0	4.9	11.4	17.8	29.7	32.7	38.1
	27	Type Ⅰ	165	6.5	13.6	23	29	0	0.7	2.5	6.9	24.5	30.9	37
		IHB	145	4.8	14.1	15	21	0	4.9	10.4	13.6	30.8	38.4	42
		Type Ⅲ	150	5.5	13.4	17	22.5	0	6.8	12.7	22.6	31.5	38.5	42.2
	30	Type Ⅰ	145	5.3	13.1	22	27	0	0.7	4	9.2	27.6	36	40
		IHB	150	5.6	13.9	16	22	0	5	13.9	17.5	30	36.5	40.7
		Type Ⅲ	155	4.5	13	18	23.5	0	5.6	16.1	22	40	43.1	48.6
10	24	Type Ⅰ	140	5.5	19.5	22	27.5	0	3.9	7.1	10.6	22	24.7	27.1
		IHB	145	5.6	17.5	15	19	0	11.3	14.3	17.1	26.8	29.1	30.1
		Type Ⅲ	160	4.8	18.4	13.5	16.5	0	15	19.5	22	29.9	33.6	34.1
	27	Type Ⅰ	145	5.3	17.9	18	24	0	5	9.3	13	24.8	25.2	29
		IHB	160	4.6	17.9	14.5	18	0	14.1	17.5	21	30	30.1	32.4
		Type Ⅲ	160	3.9	17.7	13	16	0	15.3	19.3	23	35.5	33.1	38.1
	30	Type Ⅰ	145	4.8	17.5	18.5	24	0	3.5	8.7	15.6	26.5	29.2	29.9
		IHB	150	5	17.7	14	18	0	16.2	20.5	22.7	31.2	34.4	34.8
		Type Ⅲ	145	4.1	17.2	12.5	16.5	0	20	24.5	26.1	35	39.5	41
20	24	Type Ⅰ	150	5.5	24	15	17.5	0	13	16.2	18.7	22.3	27.9	27.8
		IHB	150	4.1	23.7	10	12.5	0	18.3	21.1	21.5	29.1	32	31.1
		Type Ⅲ	150	4.6	22.7	9	11.5	2.15	24.3	26.7	27.3	33.2	35.6	36.5
	27	Type Ⅰ	160	5.1	24.2	14.5	17	0	15.2	18.6	20.3	25.4	28.6	28.3
		IHB	155	3.6	24.5	8.5	10.5	2.43	20.7	23.8	25	30.3	34.7	35.2
		Type Ⅲ	140	3.9	24.3	-	-	0	20.2	24.1	26.2	30.3	34.9	36.4
	30	Type Ⅰ	145	3.7	24.3	12	15.5	0	20.1	22.1	24.2	27.7	32.6	32.8
		IHB	145	3.9	24.1	8	10	1.8	21.8	23.7	25.8	32.3	36	38.1
		Type Ⅲ	140	3.8	23.5	8.5	11	1.39	25.1	28.7	31	37.5	38.2	39.3

**Table 6:** Statistical results for maturity and compressive strength.

Statistical Results	24 MPa			27 MPa			30 MPa		
	I	IHB	III	I	IHB	III	I	IHB	III
Correlation coefficient (R)	0.962	0.980	0.977	0.973	0.990	0.926	0.943	0.980	0.974
Determination coefficient (R^2^)	0.925	0.961	0.955	0.947	0.979	0.857	0.890	0.959	0.949
Standard error	1.79	1.24	1.97	1.72	1.17	2.52	3.10	1.68	2.32
Regression Sum of Squares	276.1	266.5	658.3	373.5	520.2	266.2	543.5	531.1	810.0
Error Sum of Squares	22.4	10.7	31.0	20.7	11.0	44.5	67.2	22.5	43.2
Total Sum of Squares	298.4	277.2	689.2	394.2	531.2	310.7	610.7	553.6	853.1
Significance F	3.5 × 10^−5^	3.4 × 10^−6^	1.1 × 10^−6^	9.9 × 10^−6^	5.0 × 10^−8^	3.4 × 10^−4^	1.3 × 10^−4^	7.5 × 10^−7^	1.8 × 10^−6^

## Data Availability

The data presented in this study are available on request from the corresponding author.

## References

[1] Yoon G.W., Lee Y.E., Baek D.H. Determination of removal time of form. Proceedings of the 2002 Korea Concrete Institute Autumn Conference.

[2] Price W.H. (1951). Factors influencing concrete strength. J. Am. Concr. Institute.

[3] Hamooni M., Maghrebi M., Majrouhi S.J., Kim S. (2020). Extending BIM interoperability for real-time concrete formwork process monitoring. Appl. Sci..

[4] Lee T., Lee J., Kim J., Choi H., Lee D. (2020). Effect of formwork removal time reduction on construction productivity improvement by mix design of early strength concrete. Appl. Sci..

[5] Vanhove Y., Djelal C. (2021). Influence of the formwork removal by polarization on the facing aesthetics in reinforced concrete. Constr. Build. Mater..

[6] Santilli A., Teixeira S., Puente I. (2015). Influence of temperature and concrete reinforcement on vertical formwork design. Constr. Build. Mater..

[7] (2022). Formwork and Copper Bars.

[8] Ahmed S., Al-Dawood J., Abed F., Mannan M.A., Al-Samarai M. (2021). Impact of using different materials, curing regimes, and mixing procedures on compressive strength of reactive powder concrete—A review. J. Build. Eng..

[9] Shi J., Liu B., Wu X., Qin J., Jiang J., He Z. (2020). Evolution of mechanical properties and permeability of concrete during steam curing process. J. Build. Eng..

[10] Peter H. (1999). Portland Cement: Classification and Manufacture. Lea’s Chemistry of Cement and Concrete.

[11] Mehta P.K., Monteiro P.J. (1993). Concrete Microstructure, Properties, and Materials.

[12] Brooks J.J., Megat Johari M.A., Mazloom M. (2000). Effect of admixtures on the setting times of high-strength concrete. Cem. Concr. Compos..

[13] Rixon R., Mailvaganam N. (1999). Chemical Admixtures for Concrete.

[14] Scrivener K.L., Juilland P., Monteiro P.J. (2015). Advances in understanding hydration of Portland cement. Cem. Concr. Res..

[15] Kim J.K., Moon Y.H., Eo S.H. (1998). Compressive strength development of concrete with different curing time and temperature. Cem. Concr. Res..

[16] Lura P., Breugel K., Maruyama I. (2001). Effect of curing temperature and type of cement on early-age shrinkage of high-performance concrete. Cem. Concr. Res..

[17] Burg R.G. (1996). The Influence of Casting and Curing Temperature on the Properties of Fresh and Hardened Concrete.

[18] Klieger P. (1958). Effect of mixing and curing temperatures on concrete strength. J. Am. Concr. Institute..

[19] Min T.B., Cho I., Park W., Choi H., Lee H. (2014). Experimental study on the development of compressive strength of early concrete age using calcium-based hardening accelerator and high early strength cement. Constr. Build. Mater..

[20] Wang C., Song M. (2021). Influence of water-cement ratio and type of mixing water on the early hydration performance of calcium sulphoaluminate (CSA) cement. Adv. Mater. Sci. Eng..

[21] Shi J., Liu B., Tan J., Dai J., Chen J., Ji R. (2020). Experimental studies and microstructure analysis for rapid-hardening cement emulsified asphalt mortar. J. Constr. Eng. Manag..

[22] Frantzis P. (2006). Effect of early-age temperature rise on the stability of rapid-hardening cement fiber composites. J. Mater. Civ. Eng..

[23] Mardani A.A., Son A.E., Felekoglu B., Ramyar K. (2017). Effect of cement fineness on properties of cementitious materials containing high range water reducing admixture. J. Green Build..

[24] (2005). Guide to Formwork for Concrete.

[25] (1993). 1993: Design Code.

[26] (2010). Execution of Concrete Structures.

[27] (2009). Korea Architectural Standard Specification Reinforced Concrete Work.

[28] (2009). Japanese Architectural Standard Specification Reinforced Concrete Work.

[29] Helal J., Sofi M., Mendis P. (2015). Non-destructive testing of concrete: A review of methods. Electron. J. Struct. Eng..

[30] Malek J., Kaouther M. (2014). Destructive and non-destructive testing of concrete structures. Jordan J. Civ. Eng..

[31] Figueira R.B. (2017). Electrochemical sensors for monitoring the corrosion conditions of reinforced concrete structures: A review. Appl. Sci..

[32] Bye G.C. (1999). Portland Cement: Composition, Production and Properties.

[33] (2017). Standard Test Method for Concrete Slump.

[34] (2021). Standard Test Method for Air Content of Fresh Concrete by the Pressure Method (Air Receiver Method).

[35] (2017). Standard Test Method for Setting Times of Concrete Mixture by Penetration Resistance.

[36] (2017). Standard Test Method for Compressive Strength of Concrete.

[37] Saul A.G.A. (1951). Principles Underlying The Steam Curing of Concrete at Atmospheric Pressure. Mag. Concr. Res..

[38] Hansen P.F., Pederson J. (1977). Maturity Computer for Controlled Curing and Hardening of Concrete Strength.

[39] Plowman J.M. (1956). Maturity and The Strength of Concrete. Mag. Concr. Res..

[40] Cario N.J. (1984). Maturity Method: Theory and Application. J. Cem. Aggreg..

[41] (2019). Standard practice for estimating concrete strength by the maturity method.

[42] Gaynor R.D., Meininger R.C., Khan T.S. (1985). Effect of Temperature and Delivery Time on Concrete Proportions. Temperature Effects on Concrete.

[43] Kim H.K., Jeon J.H., Lee H.K. (2012). Workability, and mechanical, acoustic and thermal properties of lightweight aggregate concrete with a high volume of entrained air. Constr. Build. Mater..

[44] Han C.G., Han M.C., Lee D.G. (2008). Influence of over-added AE water reducing agent on physical properties of the concrete. J. Korea Inst. Build Constr..

[45] Yi S.T., Moon Y.H., Kim J.K. (2005). Long-term strength prediction of concrete with curing temperature. Cem. Concr. Res..

[46] Schachinger I., Hilbig H., Stengel T., Fehling E. Effect of curing temperature at an early age on the long-term strength development of UHPC. Proceedings of the 2nd International Symposium on Ultra High Performance Concrete.

[47] Ayub T., Khan S.U., Memon F.A. (2014). Mechanical characteristics of hardened concrete with different mineral admixtures: A review. Sci. World J..

[48] Kim K., Park S., Moon K., Shim J. (2018). Characteristics of compressive strength of concrete due to form curing condition. KSCE J. Civ. Environ. Eng. Res..

[49] Kim G.D., Lee S.H., La W., Hwang Y.S. The practical use of early strength development technology for form stripping earlier than normal state in apartment. Proceedings of the Korea Concrete Institute Conference.

[50] Cho H.B., Kim H.Y., Lee Y.D., Jumg S.J. (2013). Effect of maintaining time of formwork on strength and dry shrinkage of mock-up concrete in cold weather condition. J. Archit. Inst. Korea Struct Constr..

[51] Li G., Luo M., Huang J., Li W. (2023). Early-age concrete strength monitoring using smart aggregate based on electromechanical impedance and machine learning. Mech. Syst. Signal Process..

[52] Kaewunruen S., Meesit R., Mondal P. (2017). Early-age dynamic moduli of crumbed rubber concrete for compliant railway structures. J. Sustain. Cem. Based Mater..

[53] Zheng Z., Wei X. (2021). Mesoscopic models and numerical simulations of the temperature field and hydration degree in early-age concrete. Constr. Build. Mater..

[54] Voigt T., Sun Z., Shah S.P. (2006). Comparison of ultrasonic wave reflection method and maturity method in evaluating early-age compressive strength of mortar. Cem. Concr. Compos..

[55] Lee S.C. (2003). Prediction of concrete strength using artificial neural networks. Eng. Struct..

[56] Yikici T.A., Chen H.L.R. (2015). Use of maturity method to estimate compressive strength of mass concrete. Constr. Build. Mater..

